# High-Throughput Approaches onto Uncover (Epi)Genomic Architecture of Type 2 Diabetes

**DOI:** 10.3390/genes9080374

**Published:** 2018-07-26

**Authors:** Anna Dziewulska, Aneta M. Dobosz, Agnieszka Dobrzyn

**Affiliations:** Laboratory of Cell Signaling and Metabolic Disorders, Nencki Institute of Experimental Biology of Polish Academy of Sciences, 02-093 Warsaw, Poland; a.dziewulska@nencki.gov.pl (A.D.); a.dobosz@nencki.gov.pl (A.M.D.)

**Keywords:** type 2 diabetes, NGS, epigenetics, GWAS, beta-cell failure, insulin resistance

## Abstract

Type 2 diabetes (T2D) is a complex disorder that is caused by a combination of genetic, epigenetic, and environmental factors. High-throughput approaches have opened a new avenue toward a better understanding of the molecular bases of T2D. A genome-wide association studies (GWASs) identified a group of the most common susceptibility genes for T2D (i.e., *TCF7L2*, *PPARG*, *KCNJ1*, *HNF1A*, *PTPN1*, and *CDKAL1*) and illuminated novel disease-causing pathways. Next-generation sequencing (NGS)-based techniques have shed light on rare-coding genetic variants that account for an appreciable fraction of T2D heritability (*KCNQ1* and *ADRA2A*) and population risk of T2D (*SLC16A11*, *TPCN2*, *PAM*, and *CCND2*). Moreover, single-cell sequencing of human pancreatic islets identified gene signatures that are exclusive to α-cells (*GCG*, *IRX2*, and *IGFBP2*) and β-cells (*INS*, *ADCYAP1*, *INS-IGF2*, and *MAFA*). Ongoing epigenome-wide association studies (EWASs) have progressively defined links between epigenetic markers and the transcriptional activity of T2D target genes. Differentially methylated regions were found in *TCF7L2*, *THADA*, *KCNQ1*, *TXNIP*, *SOCS3*, *SREBF1*, and *KLF14* loci that are related to T2D. Additionally, chromatin state maps in pancreatic islets were provided and several non-coding RNAs (ncRNA) that are key to T2D pathogenesis were identified (i.e., miR-375). The present review summarizes major progress that has been made in mapping the (epi)genomic landscape of T2D within the last few years.

## 1. Introduction

Diabetes is a multifactorial disorder that affects more than 425 million people worldwide. This number is expected to rise to 629 million cases by 2045 [1]. The disease is mostly driven by a combined effect of multiple genes, lifestyle, environmental factors, and aging. Type 2 diabetes (T2D) is the most frequent form of diabetes, comprising ~90% of all diabetes cases [1]. Type 2 diabetes develops from both the insulin resistance of peripheral tissues and dysregulation of the endocrine function of pancreatic islets-impairments in insulin release from pancreatic β-cells and greater glucagon secretion from pancreatic α-cells [2]. Consequently, uncontrollable increases in blood glucose occur as an evident but late manifestation of the presence of T2D. Type 2 diabetes is also associated with several possibly life-threatening complications, including cardiovascular disease and nephrotoxicity [3].

Complete understanding of the molecular mechanisms that are involved in the pathophysiology of T2D is a major challenge for biomedical sciences. Family-based studies have clearly shown that genetic factors play an important role in the susceptibility to T2D. The risk of the disease developing at some point of life is ~70% when both parents are diabetic and ~40% when one parent has T2D [4]. Furthermore, latest data show that more than 400 genetic risk variants at 250 loci for T2D have been identified, largely through genome-wide association studies (GWASs) [5]. Genetic predisposition and environmental factors (e.g., nutrition, gender, and age) play essential roles in the progression of T2D. Excessive exposure to fatty acids from an unhealthy diet and a sedentary lifestyle have been recognized as main causes of β-cell dysfunction and insulin resistance in skeletal muscle, which contributes to the development of T2D. An estimated 70–90% of patients with T2D are overweight or obese [6]. Epigenetic modifications that mediate the interaction between environmental factors and the genome have also been shown to be an intrinsic part of the molecular mechanism of the pathogenesis of T2D [7].

Completion of the entire human genome sequence in 2003 spawned a new era of biomedical research that seeks to understand complex diseases. The Human Genome Project and haplotype map of the human genome, spurred larger multi-institutional programs (e.g., 1000 Genomes Projects, Encyclopedia of DNA Elements [ENCODE], and Roadmap Epigenomics), that have the goal of tracking genomic and epigenomic changes across multiple populations [8]. Aforementioned studies enabled GWASs for complex diseases such as T2D. DNA amplification, Sanger sequencing, and microarray studies have shed light on the genetics of diabetes but have only provided a limited amount of data. An emerging, cost-effective approach to high-throughput T2D research that is based on massive, parallel sequencing detection methods is next-generation sequencing (NGS). Next-generation sequencing, combined with microarray-based methods, provides powerful tools for large-scale genomic and epigenetic research of T2D. Such approaches include chromatin immunoprecipitation followed by sequencing of targets (ChIP-seq), RNA sequencing (RNA-seq), methylation site mapping (methyl-seq), and copy number variant detection (CNV-seq). As the possibility to sequence single molecules has become a reality, third-generation sequencing (TGS) will likely also be achieved soon. The increase in the number of available NGS and TGS platforms will also further decrease costs and increase efficiency of sequencing in both basic and clinical studies of T2D. To date, NGS technology has provided an extensive increase in the amount of convincing data, confirmed by different epigenetic studies, that provide a broader insight into the molecular bases of T2D [9]. The present review summarizes major contributions of NGS-related techniques to defining the (epi)genomic landscape of T2D within the last few years.

## 2. Genome-Wide Association Studies of Type 2 Diabetes

During the past decade, GWASs that emerged after the human genome sequence was completed enabled substantial progress in elucidating the genetic basis of T2D [8]. Before these GWASs, genetic linkage analyses and candidate gene studies were the primary methods that were used to establish links between genotypes and phenotypes of common diseases. These methods successfully identified genes that cause monogenic variants of T2D, such as maturity onset diabetes of the young (MODY). However, when applied to more common, complex forms of T2D, these methods could recognize only a few T2D-linked genes that had the strongest effect (e.g., *TCF7L2*, *PPARG*, and *KCNJ11*) [10]. Genome-wide association studies validated these “old culprits” of T2D and expanded them to include hundreds of single-nucleotide variants (SNVs) that represent more than 150 genomic loci that are associated with T2D, insulin secretion, and insulin resistance [11]. Besides *TCF7L2*, *PPARG*, and *KCNJ11* loci, the most replicated T2D susceptibility variants identified in GWASs were found in and around *CDKN2A/2B*, *IGF2BP2*, *SLC30A8*, *CDKAL1* and *FTO* genes [12,13,14,15]. The variants that are most strongly associated with T2D are preferentially located at active enhancers in pancreatic islets and to a lesser extent at enhancers that are active in tissues that are key to insulin action, i.e., adipose tissue, muscle, and liver [12,13,14,15]. The extension of GWASs beyond array-based genotyping to assess a broader range of low-frequency variants revealed that genetic variations that influence T2D appear to reside mostly at common variant sites [16]. Nonetheless, the loci that are identified by GWASs explain only a fraction (<20%) of T2D heritability [17].

### 2.1. Discovery of Genetic Variants Associated with Type 2 Diabetes 

The first GWAS of T2D was based on 661 cases of T2D and 614 non-diabetic controls from France using the Illumina platform, which found two regions (*HHEX* and *SLC30A8*) that were novel T2D susceptibility loci [18]. Soon afterward, three collaborating groups: Wellcome Trust Case Control Consortium (WTCCC), Finland-United States Investigation of NIDDM Genetics group (FUSION), and Diabetes Genetics Initiative (DGI) replicated previous GWAS results and independently discovered additional associations at *CDKAL1*, *IGF2BP2*, and *CDKN2A/B* [12]. Investigators from WTCCC, DGI, and FUSION ultimately joined forces and combined their data to form the Diabetes Genetics Replication and Meta-Analysis (DIAGRAM) Consortium, which performed the first GWAS meta-analysis of T2D. As a result of this collaboration, six novel T2D susceptibility loci were discovered (mapping near *NOTCH2*, *CDC123/CAMK1D*, *THADA*, *JAZF1*, *TSPAN8/LGR5*, and *ADAMTS9*) [13]. The formation of a large international consortium to meta-analyze summary-level data marked an important progression from studies at the cohort level that enabled a substantial increase in sample size, an increase in statistical power to detect associations, and the ability to uncover common variants for T2D with a low effect size. To date, the DIAGRAM consortium has submitted the largest GWAS dataset for samples of European descent (12,171 cases of T2D and 56,862 non-diabetic controls). This dataset was subsequently used as a basis for an expanded meta-analysis of SNVs on the Metabochip, including approximately 150,000 individuals (34,840 cases and 114,981 controls). Genome-wide analyses of these data confirmed common variant signals and added to the list another 10 loci of T2D susceptibility, including two that demonstrated sex-differentiated associations [19]. The most recent meta-analysis of genome-wide association data from 26,676 T2D cases and 132,532 controls of European descent after imputation was performed using the 1000 Genomes multiethnic reference panel. This analysis identified 13 novel T2D-associated loci (including variants near the *GLP2R*, *GIP*, and *HLA-DQA1* genes) and brought the total number of independent T2D associations to 128 distinct signals at 113 loci [20].

The vast majority of the most common variant sites that are related to T2D were identified by GWASs using large case-control cohorts of European descent. However, because of the specificity of common SNVs across major ethnic groups and the greater number of diverse populations that are genotyped, more specific variants have been identified [21]. The identification of *KCNQ1* in East-Asian samples, which was not previously detected in samples of European descent, illustrated the possibility of uncovering additional T2D loci from different populations [22]. Additionally, in populations of Asian descent, novel associations have been found that reach genome-wide significance at *PAX4* [23], *SRR*, *PTPRD*, *UBE2E2* and *CDC4A-CDC4B* [24]. Population-specific genetic variants associated with T2D susceptibility were also identified in American Indians (DNER) [25], Inuits (*TBC1D4*) [26], and Sikhs from India (*SGCG*) [27].

### 2.2. From Genome-Wide Association Studies to Biological Function and Translational Medicine

A large number of genetic loci that are associated with T2D susceptibility have been identified in GWASs. However, only little progress has still been made in moving from association signals towards functional transcripts and understanding their precise role in the development of T2D. Nevertheless, some significant advances have been made as a direct result of GWASs, leading to the elucidation of novel disease-causing pathways and translational medicine applications. Currently, it is possible to assign compelling effector transcripts to approximately one-third of T2D loci that have been identified by GWASs [16,28,29]. A wide variety of network-based approaches have been applied to investigate the extent to which the genetics of T2D predisposition converge on a restricted set of biological pathways. Several T2D risk variants have been identified as primary regulators of insulin secretion, insulin action, and pancreatic islet transcription factors. [10,16]. The newly discovered SNVs allow the better characterization of abnormalities in early insulin processing and secretion. *TCF7L2*, *SLC30A8*, *C2CD4B*, and *GIPR* were associated with higher proinsulin secretion and lower insulin secretion [30]. In the same study, SNVs found in transcripts of *MTNR1B*, *FADS1*, *GCK*, and *DGKB* were associated with a lower insulinogenic index [30]. Data from human islets have characterized likely effector transcripts at several loci where their major impact is to reduce insulin secretion, such as *ZMIZ1*, *MTNR1B*, and *ADCY5* [16,31]. Risk variants of *CDKAL1* were associated with defects in insulin secretion, with impairments in the insulin response in glucose tolerance tests [32]. Additionally, functional variants of *GCKR* (which may increase hepatic glucose production) and *IGF1* (which stimulate glucose transport into adipose tissue and muscles) were found to influence insulin sensitivity [9]. Cis-expression mapping has highlighted the association between adipose tissue-specific *KLF14* signaling and insulin resistance and hyperlipidemia [10]. Potential causal variants at *JAZF1* and *CDC123*/*CAMK1D* loci were also identified that appear to act as part of cis-regulatory elements that affect the binding of *PDX1* and *FOXA1/FOXA2* transcription factors, respectively [29]. Moreover, GWAS offered a broader insight into the epidemiology of T2D by identifying potential genetic links between lipid dysregulation and glycaemia (*FADS1*, *HNF1A*, and *GCKR*), circadian rhythmicity and metabolic alterations (*MTNR1B* and *CRY2*), and between low birth weight and subsequent T2D risk (*ADCY5*) [10,16].

Better mapping of the genetic landscape of T2D that has been provided by GWASs has opened new avenues for translational medicine and drawn attention to potential targets for pharmacological interventions. The *SLC30A8* gene, which encodes the zinc transporter ZnT-8 that is expressed in pancreatic β-cells, is one of the earliest and most illustrative examples of the use of T2D-associated SNVs in anti-diabetic therapy. Loss-of-function mutations at *SLC30A8* have been shown to be protective against T2D, which has led several pharmaceutical companies to develop ZnT-8 antagonists [33]. In pharmacogenetic studies, the impact of genetic variants on the response to commonly used therapeutic agents extends our knowledge of the mechanisms through which these agents operate. GWASs provide the opportunity to mine the entire genome for common variants that are associated with the therapeutic response to metformin (*SLC22A1*, *SLC47A1*, *ATM*, and *SLC2A2*), sulfonylurea group compounds (*TCF7L2*, *KCNJ11* and *ABCC8* [34]. Finally, genetic variants were also used to assess relationships between circulating lipid levels, early nutrition, vitamin D intake, and chronic inflammation and the risk of T2D [16].

Identifying SNVs that are associated with T2D using large-scale GWASs and meta-analyses has undoubtedly been a tremendous success. However, the common variants that have been uncovered by GWASs have only contributed modestly to explaining T2D heritability. Rare- or low-frequency functional genetic variants and epigenetic control might explain to a significant extent the “missing” T2D heritability. 

## 3. Uncovering the Significance of Rare-Coding and Non-Coding Genetic Variants in the Etiology of Type 2 Diabetes

As previously stated, GWASs have uncovered many new genetic associations that are relevant to T2D, but GWAS findings represent common and mid-frequency genetic variations, thus excluding rare frequency variants and also cumulative effect of many variants with small effect sizes. “Missing heritability” refers to the portion of genetic variance that cannot be explained by all significant single-nucleotide polymorphisms (SNPs). This discrepancy might partly result from incomplete linkage between causative genetic variants and those genotyped, or due to rare genetic variants [35]. It has been suggested that missing T2D heritability might be explained by combined action of many variants, that drive T2D susceptibility and are aggregated in molecular pathways that govern metabolic traits important for insulin/glucose action in certain tissues [36]. The power of GWAS to identify a true association between a SNP and these traits depends on the phenotypic variance, which is determined by the frequency of variants in the sample and how strongly they differ in their phenotypic effect. Thus, GWAS fails to identify rare-variants that account for a large effect on the phenotype. Several important considerations on how to overcome fundamental limitations of GWAS and increase the power of the study (epistatic interactions such as incomplete genotyping or genetic heterogeneity) were issued. The proposed solutions include increasing the sample size by e.g., mixed model setting [37] or statistical models that analyze correlated traits and still correcting for population structure [33].

Next-generation sequencing and array-based techniques have allowed an increase in statistical power and the identification of rare and non-coding genetic variants that are associated with diseases. Whole genome sequencing (WGS) allows complete DNA sequencing and therefore provides data on structural, rare and de novo mutations in the noncoding genome that might contribute to disease etiology. Whole exome sequencing (WES) is a cost effective alternative which limits the sequencing to genomic fraction that encodes for messenger RNA (mRNA), and is therefore sufficient to explain the molecular basis of genetic variation. Several NGS platforms are available, each of which has a different read length, runtime, and error rate. The most common is the Illumina platform, which utilizes sequencing-by-synthesis and paired-end sequencing methods that allow easy data assembly [38]. Pacific Bioscience and Helicons developed platforms that enable the longest reads (typically 10 kb) without needing to amplify the template. Diligent research designs increase the statistical power of studies, including extreme phenotypes, isolated populations, and familial sampling allow to identify rare variants, previously “missed” by GWAS limitations. Currently, rare variants are defined as those that cannot be detected individually but need to be tested in large sample set, and can be present in one or only a few samples [39]. Rare-coding and non-coding genetic variants significant in the pathogenesis of T2D are described in further paragraphs and were summarized in Table 1.

### 3.1. Low-Frequency Genetic Variants vs. Population Risk

Genetic origin is an important factor in identifying disease risk alleles. Many high-throughput approaches have led to the identification of disease risk variants that are common in the population of interest but rare in other populations. The SIGMA Type 2 Diabetes Consortium analyzed 9.2 million SNPs in Latin Americans (3848 diagnosed with T2D and 4366 matched controls) and identified a novel risk variant with four amino acid substitutions in *SLC16A11* that was present in 50% of Native American cases of T2D. The SLC16A11 protein is localized in the endoplasmic reticulum and plays an important role in lipid metabolism in liver, salivary gland, and thyroid [45]. Notably, this variant is very rare in European and African samples [45]. In the Latin American population, a low-frequency variant of the *HNF1A* gene that is associated with T2D was identified by whole-exome sequencing [40]. Another study was conducted with an unrelated Chinese population (384 cases of T2D and 1468 controls), and six novel SNP rare-coding variants were identified (rs35264875, rs267603153, rs267603154, rs3829241, rs1551305, and rs3750965) using the Sequenom MassARRAY SNP genotyping system [41]. *TPCN2* encodes lysosomal two-pore channel 2, which is thought to be a novel gene that is important for regulating insulin and glucose homeostasis [46,47,48]. The authors identified a variant of *TPCN2* (rs1551305) that was associated with T2D [41]. This finding revealed new traits in diabetes research. Further work is needed to elucidate the underlying mechanisms of *TPCN2*’s actions. The outcome of such studies clearly highlights the need for the personalization of population-based treatment.

Extreme phenotype sampling (150,000 individuals of Northern Europe ancestry at the extremes of T2D risk) allowed identification of a rare nonsense variant of *SLC30A8* that protects against T2D using Illumina HiSeq 2000 [33]. In another study, a WGS was performed with 2630 Icelanders, followed by testing on Iranian and Danish populations. This study revealed four new rare variants that affect the prevalence of T2D. A rare (0.20%) frameshift variant of *PDX1* (a crucial pancreatic β-cell identity gene) was identified and correlated with a higher risk of developing T2D. Two rare missense variants (4.98% and 0.65% frequencies) of the *PAM* gene were associated with a higher risk of T2D. Conversely, the presence of a *CCND2* variant (rs76895963; 1.47% frequency) in intron 1 protected against T2D and was correlated with higher *CCND2* expression [42]. 

In a recent study of the European population, array-based genotyping at 2.5 million SNVs was performed in an effort to identify rare variants that are associated with T2D [23]. However, no novel rare variants that were exclusive to T2D were found in multi-ethnic samples either within coding regions or in non-coding regulatory elements. Consistent results were obtained in another deep WGS analysis (40× coverage) of 1034 samples from 20 large Mexican-American families with a high prevalence of T2D. These authors found rare variants that were not detected previously in population studies, but none of them were associated with T2D [49]. Larger multi-population studies and more advanced study methods are needed to reliably identify rare variants that are exclusively associated with T2D to eventually uncover “missing” T2D heritability. 

### 3.2. Genetic Variants in Familial Studies of Type 2 Diabetes

The development of T2D is driven by the combined effect of environmental factors and a strong hereditary component. Estimates for the heritability of T2D range from 20–80% and an evidence for heritability comes from variety of population, family-based and twin-based studies [24]. To date, the causative genetic variants identified in GWAS have explained only a small proportion of T2D heritability. 

The Botnia study was conducted with more than 1400 Finnish and Swedish families (11,000 participants) and found that the heritability of T2D strongly depended on the age of the population and peaked in middle age (35–60 years old). The authors estimated that sibling risk (λs) increased to 8 for siblings with a family history of T2D compared with siblings from unrelated population [50]. To validate the data, SNPs that showed a parent-to-origin effect (POE) on T2D were analyzed using samples that were obtained from another genotyped population (the family-based Hungarian Transdanubian Biobank, HTB). In both populations, the rs7578597 SNP of the *THADA* gene was found, showing excess transmission of the maternal risk T allele to diabetic offspring. Furthermore, SNP variants for loci in the *KCNQ1* (rs163184) gene, *TCF7L2* (rs7903146) gene, and a region near the *ADRA2A* (rs10885122) gene were associated with the development of T2D in both populations [43].

As an example of the familial inheritance of T2D within environmentally isolated populations, another study was performed among Pima tribe members with a high risk of T2D. Whole-exome sequencing was performed with 177 Pima Indian samples, and 345 SNP calls were identified and genotyped in follow-up studies. Of the variants that were identified, *CYB5A* (rs7238987) and the newly identified *RNF10* variant had associations with T2D and obesity that reached genome-wide significance [44]. The authors concluded that the *RNF10* variant might be specific to the Pima tribe. Notably, *CYB5A* encodes cytochrome b5 type A, a microsomal hemoprotein that acts as an electron carrier for stearoyl-CoA desaturase (SCD). This enzyme catalyzes the conversion of saturated fatty acids to monounsaturated fatty acids, and its alterations are associated with obesity-related disorders, including T2D [51].

## 4. Single-Cell RNA-seq as a Novel Approach in High-Throughput Type 2 Diabetes Research

Islets of Langerhans are heterogeneous structures that consist of different cell types. Further research is needed to track genetic changes in individual pancreatic islet cells and in sorted cell populations. The massive development of NGS allowed the sequencing of single cells from human pancreatic islets. Considering the cell-type heterogeneity within Langerhans islets, such an approach may reveal novel T2D susceptibility loci. RNA-seq that was performed with single cells (*n* = 1492) from T2D pancreatic islets (*n* = 6) and healthy donors (*n* = 12) identified 245 genes that were associated with T2D. The function of 28% of the identified genes is still unknown [52]. Interestingly, according to these authors, almost 40% of the affected genes were associated with cell growth in non-islet cells. These data support the widely accepted notion that lower β-cell mass contributes to the progression of T2D [53]. Moreover, single-cell sequencing allowed the identification of genes that are specific to human α-cells (*GCG*, *DPP4*, *FAP*, *PLCE1*, *LOXL4*, *IRX2*, *TMEM236*, *IGFBP2*, *COTL1*, *SPOCK3*, and *ARRDC4*), β-cells (*INS*, *ADCYAP1*, *IAPP*, *RGS16*, *DLK1*, *MEG3*, *INS-IGF2*, and *MAFA*), δ-cells (*SST*, *BCHE*, *HHEX*, and *RPL7P19*), and PP cells (*PPY*) [52]. 

To identify cell type-specific changes in expression, single islet cells that were dissected from T2D individuals and non-diabetic controls were compared. Among the 410 genes that presented differential expression in T2D samples compared with controls, 248 were of β-cell origin, 138 were of α-cell origin, and 28 were differentially expressed from δ-cells. Moreover, 74 genes of acinar origin, 35 genes of ductal origin, and 28 genes of stellate exocrine origin were identified. These data clearly imply cross-talk between endocrine and exocrine cell types in the pathogenesis of T2D [54]. 

## 5. Genome-Wide Profiling of Epigenetic Changes in Pancreatic Islets and Peripheral Tissues

Epigenetic data added another layer of complexity to our understanding of the genomic bases of T2D. Given that a variable epigenetic pattern can modulate the link between the SNP and trait, consideration of this interplay is critically important. Molecular epigenetics involves changes in gene function that occur without a change in the nucleotide sequence via DNA methylation, histone post-translational modifications (PTMs), or non-coding RNA (ncRNA) [7]. Epigenetic modifications that appear as direct covalent modifications of histone core proteins, DNA, or RNA can lead to reinterpretation of the genomic DNA sequence and the activation/repression of specific gene sets within each cell type and consequently affect the function of these cells [55,56]. The majority of studies of associations between T2D and epigenetic alterations have been performed among Chinese, Swedish, and American populations. These studies assessed epigenetic signatures, namely global and gene specific DNA methylation and histone modifications, in blood, pancreatic islets and insulin-sensitive tissues [57]. Overall, data on global and regional DNA methylation and histone modifications that are related to T2D that have been generated to date have been derived from over 10,800 unique participants, with more than 3300 T2D cases [57]. Bisulfite conversion followed by NGS and DNA methylation arrays is a versatile tool that has significantly contributed to the development of successful projects, such as ENCODE and Roadmap Epigenomics. The outcomes of these projects, such as the Epigenome Atlas and National Center for Biotechnology Information Gene Expression Omnibus repositories, are publicly available databases that comprise functional maps of epigenetic markers that have been annotated for various human tissues (including those that are related to T2D development) and model organisms. The combination of data from high-throughput approaches and association studies has provided compelling evidence that some epigenetic markers contribute to the risk of T2D [57,58]. Epigenetic alterations have been shown to affect the expression of genes that are crucial for maintaining pancreatic islet secretory capacity, survival, and functional identity and the proper response to insulin in peripheral tissues [59,60]. Furthermore, several epigenetic signatures, such as circulating microRNAs (miR-375 and miR-126) [61], differentially methylated circulating DNA in insulin gene promoter [62], and histone deacetylase 3 (HDAC3) inhibitors [63] have been proposed as novel tools for T2D diagnosis [61], prediction [62], and treatment [63], respectively. 

### 5.1. DNA Methylation Signatures in Type 2 Diabetes

To date, DNA methylation has been the most widely studied among epigenetic players in the context of T2D. The first reports of the contribution of DNA methylation to T2D development came from a study of individuals who suffered from the Dutch famine during prenatal development. Malnutrition resulted in a higher risk of developing T2D in elderly individuals and offspring of mothers who were exposed to famine during pregnancy [64]. Detailed analyses revealed changes in methylation patterns within promoter regions of genes that are related to metabolic and cardiovascular disorders (*IGF2*, *GNASAS*, *IL10*, *LEP*, *ABCA1*, *INS-IGF2*, and *MEG3*), and these changes were associated with prenatal malnutrition [64]. 

Initial studies that were performed on pancreatic islets were candidate-driven and indicated a correlation between lower mRNA expression and excessive promoter hypermethylation of *INS* (encoding insulin), *PDX1* (encoding a transcription factor that is important for both pancreatic development and the function of mature β-cells), *GLP1R* (encoding the GLP1 receptor that stimulates insulin secretion), and *PPARGC1A* (encoding the mitochondrial regulator peroxisome proliferator-activated receptor γ coactivator 1α [PGC1α]) in islets from T2D donors [55]. The first genome-wide DNA methylation study of human pancreatic islets was performed using the Infinium HumanMethylation27k BeadChip (which covers ~27,000 CpG sites, representing ~0.1% of the CpG sites in the entire human genome). This study revealed 254 genes with differential DNA methylation in T2D islets [65]. Currently, the Infinium HumanMethylation450K BeadChip (which covers ~480,000 CpG sites, representing ~1.7% of the CpG sites in the entire human genome) remains the most commonly applied array for genome-wide methylation analyses. The analysis that was performed with the Infinium 450K array extended to 853 the number of genes that display significantly different DNA methylation patterns within promoter regions in islets from T2D donors [66]. Although methylation arrays have greatly improved the analysis of DNA methylation, they are still capable of verifying only a small proportion of CpG sites in the genome. Recently, whole-genome bisulfite sequencing (WGBS) was performed to obtain a more complete picture of the diabetic islet methylome. WGBS is the most comprehensive and unbiased method to study DNA methylation at single-nucleotide resolution. In the WGBS study, ~83% of all CpGs in the islet genome was covered, and 25,820 differentially methylated regions (DMRs) in T2D islets compared with non-diabetic islets were identified. Additionally, 475 genes (including *SLC2A2*, *NR4A3*, *PARK2*, *SOCS2*, and *PID1*) were found that exhibited both DMRs and significant changes in expression in T2D islets [67].

One of the master metabolic switches in skeletal muscle is PGC1α, which is encoded by the *PPARGC1A* gene that was identified in early linkage studies as one on the top hits with regard to T2D pathogenesis. Based on a combination of methylated DNA immunoprecipitation and an Affymetrix promoter array approach, 838 differentially methylated promoter regions were found in samples from T2D patients compared with healthy controls. The authors found that higher *PPARGC1A* promoter methylation was associated with a decrease in gene expression in samples from T2D donors [68]. In another study, Infinium 27 K arrays were used to analyze samples from skeletal muscle biopsies that were taken from monozygotic twins who were discordant for T2D. Variations in DNA methylation between diabetic and non-diabetic twins in promoter regions of *IL8*, *CDKN2A*, *DUSP9*, *HNF4A*, *HHEX*, *KCNQ1*, *KLF11*, *PPARGC1A*, and *SLC30A8* were found. In the same study, alterations of DNA methylation in subcutaneous adipose tissue were found in T2D twins in promoter regions of the following genes: *ADCY5*, *CAV1*, *CIDEC*, *CDKN2A*, *CDKN2B*, *DUSP9*, *HNF4A*, *IDE*, *IRS1*, *KCNQ1*, *MTNR1B*, *TSPAN8*, and *WFS1* [69]. Another gene that is crucial for skeletal muscle homeostasis is *PDK4*, which encodes pyruvate dehydrogenase kinase 4 that is involved in glucose and lipid metabolism. Methylation of the *PDK4* promoter was lower in T2D and inversely correlated with *PDK4* gene expression. Furthermore, *PDK4* expression was positively correlated with Body Mass Index (BMI), blood glucose, insulin, C peptide, and glycated hemoglobin levels [70]. The distinct methylation patterns that were observed in peripheral tissues as a response to obesity or acute weight loss should be considered in prospective studies.

### 5.2. Chromatin Modification Profile 

To gain further insights into coordination of the epigenome in T2D, the relationship between DNA methylation and other epigenetic markers (e.g., histone modifications and ncRNA) have been studied across the genome. The first profile of chromatin accessibility in human pancreatic islets was performed using formaldehyde-assisted isolation of regulatory elements coupled with high-throughput sequencing (FAIRE-seq). Approximately 80,000 open chromatin sites and 340 genes (e.g., β-cell expression of *PDX1*, *SLC30A8*, and *NKX6*.1 loci) with islet-selective open chromatin regions were identified [71]. More recently, the assay for transposase-accessible chromatin with high-throughput sequencing (ATAC-seq) has been successfully used to provide more detailed maps of open chromatin in sorted human α- and β-cells [72]. The integration of high-resolution maps of islet-specific histone modifications with WGBS data uncovered correlations between the level of DNA methylation, histone modifications, and chromatin accessibility. The lowest degree of DNA methylation was detected in regions that were occupied by histone modifications that were associated with open chromatin (11.8% for H3K9ac and 7.8% for H3K4me3). Higher methylation levels revealed regions that were occupied by modifications that were associated with repressive chromatin (49.6% for H3K27me3 and 80.2% for H3K9me3) [67]. Several studies also indicated that histone deacetylase 5 (HDAC5) plays a role in insulin’s actions in skeletal muscle by influencing H3 acetylation levels, thereby modifying the expression of *PPARGC1A* and *GLUT4* [73].

### 5.3. The Role of ncRNA in Type 2 Diabetes Pathogenesis

In addition to DNA methylation and histone modifications, ncRNA is another important player in the epigenetic control of gene expression. Most transcripts with protein-coding potential that have been identified by high-throughput studies can be regulated by miRNAs (i.e., short RNA molecules, 17–21 nucleotide length) that are involved in transcriptional and post-transcriptional gene silencing [61,74]. The first catalog of miRNAs in pancreatic islets was established using Illumina small RNA-seq. A total of 366 unique miRNAs were found in pancreatic islets, 40 of which were islet-specific (the islet-cell miRNA profile was compared with miRNA profile of 15 other human tissues including liver, adipose tissue, skeletal muscle, heart, kidney, lung, skin) [74]. Afterward, ultra-high-throughput RNA-seq allowed the identification of more than 800 miRNAs that were expressed in human pancreatic islets from non-diabetic and T2D donors [75]. Gradually, miR-124-3p, miR-129-3p, miR-187-3p, miR-187-5p, miR-224-5p, miR-345-5p, miR-375, and miR-589-5p (which exhibit higher expression in T2D islets compared with non-diabetic islets) and miR-136-5p, miR-369-3p, miR-411-5p, miR-432-5p, miR-487a-3p, miR-487b-3p, miR-495-3p, miR-539-3p, miR-655-3p, miR-656-3p, and miR-7-5p (which exhibit lower expression in T2D islets compared with non-diabetic islets) have been classified into a group of miRNAs that are the most dysregulated in the case of T2D [61]. In skeletal muscle of T2D patients, higher expression of let-7f-5p was shown to be associated with the lower expression of *INSR* and *IRS2*. In another study, the lower expression of miR-15b-5p in human T2D skeletal muscle was associated with the lower expression of *IRS1* and *INSR* [76]. Other studies found that higher levels of miR-206 and lower levels of miR-133a-3p were related to the pathogenesis of T2D, although target loci remain unknown [77]. 

**Figure 1:**
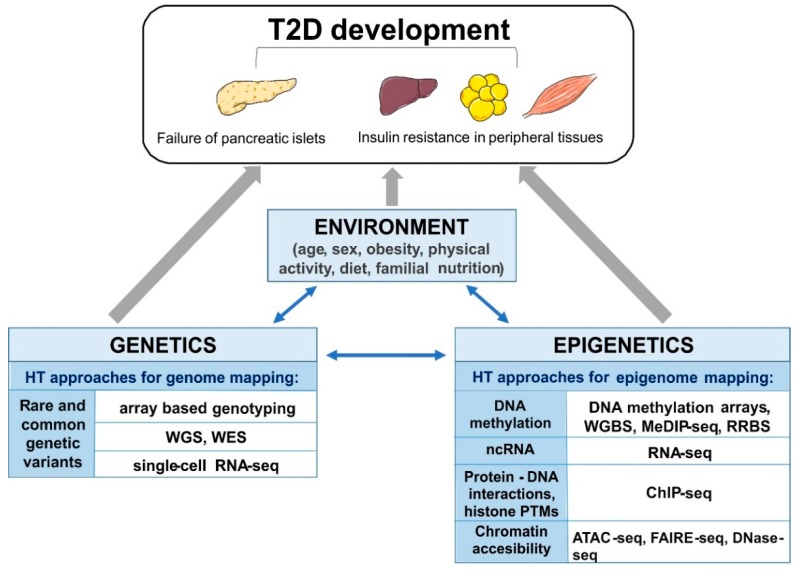
The schematic overview of the interplay of mechanisms involved in development of type 2 diabetes (T2D), and high-throughput (HT), next generation sequencing (NGS) approaches applied to study (epi)genetic modifications. Whole genome-seq (WGS), whole exome-seq (WES), RNA-sequencing (RNA-seq), single-cell RNA sequencing (single-cell RNA-seq), whole genome bisulfite sequencing (WGBS), reduced representation bisulfite sequencing (RRBS), methylated DNA immunoprecipitation sequencing (MeDIP-seq), chromatin immunoprecipitation-sequencing (ChIP-seq), Assay for Transposase-Accessible Chromatin using sequencing (ATAC-seq), Formaldehyde-Assisted Isolation of Regulatory Elements (FAIRE-seq) and DNase I hypersensitive sites sequencing (DNase-seq).

Emerging evidence highlights the role of long non-coding RNAs (lncRNAs; >200 nucleotide length) in the epigenetic control of T2D progression and its associated complications [78]. The integration of sequence-based transcriptome and chromatin maps of human islets allowed the identification of 1128 islet lncRNA genes. This study also found two lncRNAs (KCNQ1OT1 and HI-LNC45) that were differentially expressed in islets from T2D donors [78]. Altogether, these data suggest that ncRNAs and other epigenetic markers might be useful tools for the diagnosis, prevention, and treatment of T2D. The above mentioned high-throughput approaches are summarized schematically in Figure 1. 

### 5.4. Epigenome-Wide Association Studies of Type 2 Diabetes

Since the array based methods for DNA methylation analyses have been introduced, the focus turned to epigenome-wide association studies (EWASs). Ongoing EWASs have progressively defined links between epigenetic control, candidate genetic variants that are identified in GWASs, and their target genes. Epigenome-wide association studies performed to date, despite limitations in detecting tissue-specific alterations and the requirement to correct for cell composition heterogeneity, have reported over 50 unique CpGs for T2D in peripheral blood and changes in DNA methylation in different tissues, including the pancreas (15 CpGs), adipose tissue (10 CpGs), and liver (two CpGs) [79]. The first EWAS was performed to search for T2D-related DNA methylation variations in peripheral blood. This study found that known GWAS loci are enriched with differentially methylated sites. The study also found that the hypomethylation of a CpG site in the *FTO* gene was significantly associated with T2D risk [56]. Additionally, the distribution of T2D-associated DMRs in pancreatic islets indicated that 55% of the DMRs were located within 1501–50,000 bp upstream from the transcription start site (TSS), 1.5% of the DMRs were located within 201–1500 bp upstream from the TSS, and 1.0% of the DMRs were located within 1–200 bp upstream from the TSS. Furthermore, 12.5% of the DMRs were located within transposable elements. The examination of overlap between T2D-associated islet DMRs and 65 T2D candidate genes that were identified in a GWAS revealed that 159 DMRs were annotated to 43 known candidate genes for T2D. Of these, T2D susceptibility genes with the highest number of DMRs were *TCF7L2*, *GLIS3*, *THADA*, and *KCNQ1* [67]. Another longitudinal EWAS (based on the measurement of methylation in the same individual during life) of T2D identified five CpGs (in *TXNIP*, *ABCG1*, *PHOSPHO1*, *SOCS3*, and *SREBF1* loci) that were associated with T2D onset in Indian Asians [80]. Recent EWASs also reported that differentially methylated CpGs, which are both age-related and T2D-specific, can appear in parallel in blood and other tissues. In pancreatic islets, ~60% of the methylation alterations that were associated with age, including methylation changes in genes that are known to be associated with T2D (e.g., *FAM123C*, *KLF14*, *FHL2*, and *GNPNAT1*), were also present in blood. Furthermore, differentially methylated CpGs in the *SOCS3* locus in the liver, *SREBF1* gene in pancreatic islets, and *TXNIP* locus in the liver, pancreatic islets, and skeletal muscle were also reflected in blood [81]. 

The integration of DNA methylation data (WGBS) and chromatin accessibility data (ATAC-seq) with established ChIP-seq markers have provided an opportunity to create high-resolution chromatin state maps in pancreatic islets. According to these detailed epigenome maps, GWAS signals for T2D are enriched in subsets of islet enhancers: strong enhancers, marked by both H3K4me1 and H3K27ac, weak enhancers marked by H3K4me1 only, gene enhancers marked by H3K4me1 and H3K36me3, characterized by hypomethylation and open chromatin [82]. To further explore the functional importance of certain SNPs in T2D susceptibility, a recent study by Sun et al. integrated GWAS data and cell/tissue-specific histone modification ChIP-seq data (27 T2D-relevant cell/tissue types) to identify T2D-associated SNPs in super enhancers (i.e., clusters of transcriptional enhancers that are located in protein-non-coding regions). A total of 286 potentially functional T2D super-enhancer SNPs were found. Interestingly, 57 of these super-enhancer SNPs exhibited strong regulatory potential, including 20 SNPs that are involved in regulating chromatin interactions, four SNPs that are involved in regulating lncRNA, and four SNPs that overlap with CpG islands [83].

The largest EWAS that has been performed to date analyzed skeletal muscle biopsies of the *vastus lateralis* from 271 Finnish individuals (with normal and impaired glucose tolerance, impaired fasting glucose, or newly diagnosed T2D) and applied a combination of high-throughput methods. Deep RNA-sequencing and dense genotyping data were integrated with epigenome data, including ATAC-seq [84]. Muscle-specific genomic traits for T2D were identified that regulate the transcriptional activity of several genes, including *ANK1*, which is enriched with SNPs that are located within a super-enhancer region (>3 kb from the TSS). *ANK1* isoforms were recently reported to be associated with sarcoplasmic reticulum assembly, which is crucial for *GLUT4* translocation to the plasma membrane and insulin-stimulated glucose uptake [85]. Moreover, *ANK1* is thought to interact with IRS1, another critical point in the insulin signaling cascade in skeletal muscle [86]. 

Obesity is a key risk factor for the development of T2D. To find potential associations between DNA methylation and BMI, an EWAS that included 5387 individuals from European and Indian Asian populations was performed. In blood samples, 278 CpG sites were identified that were strongly associated with BMI with epigenome-wide significance. Of the 298 CpG sites, 187 were subsequently replicated. Interestingly, comparisons of CpG sites within blood and peripheral tissues (i.e., white adipose tissue, liver, skeletal muscle, and pancreas) found that the mean methylation pattern of 187 CpG sites was similar among all of the analyzed tissues. These data strongly support the hypothesis that the observed level of DNA methylation is a consequence of adiposity [87]. Furthermore, based on the proximity of methylation markers to the nearest gene and functional genomics, the authors identified 210 candidate genes that are involved in the association between BMI and DNA methylation. Gene set enrichment analyses revealed that many of the identified genes (e.g., *ABCG1*, *LPIN1*, *HOXA5*, *LMNA*, *CPT1A*, *SOCS3*, *SREBF1*, and *PHGDH*) participate in the development of insulin resistance and lipid metabolism [87]. Moreover, the combined effect of a short-term high-fat diet and resistance exercise followed by genome- and epigenome-wide profiling was examined. Significant changes in DNA methylation were associated with gene expression in both groups. Exercise did not prevent the inflammatory process that was induced by the high-fat diet but provoked muscle adaptation and protected against muscle atrophy [88]. 

The authors adapted several sophisticated approaches, such as micrococcal nuclease digestion followed by high-throughput sequencing (MNase-seq) to map the genomic location of histones, RNA-seq to identify small ncRNA, and reduced bisulfate sequencing on a MiSeq Illumina Instrument to analyze CpG methylation sites and SNP calls in genomic DNA using the Infinium CoreExome-24 BeadChip (Illumina), in lean and obese participants before and after gastric bypass surgery. Similar histone positioning and marked differences in DNA methylation and small ncRNA expression were observed in gametic cells that were obtained from obese participants before and after acute weight loss The remodeling of DNA methylation patterns occurred at loci that are associated with appetite control, such as the melanocortin 4 receptor (MC4R), brain-derived neurotrophic factor (BDNF), neuropeptide Y (*NPY*), cannabinoid receptor type 1 (*CB1*), and cocaine and amphetamine regulated transcript (*CART*). Furthermore, changes in CpG methylation were observed in genes that are related to obesity and metabolism, including fat mass and obesity associated protein (*FTO*), carbohydrate sulfotransferase 8 (*CHST8*), and SH2 binding domain-containing protein 1 (*SH2B1*) [89]. Table 2 summarizes currently annotated T2D associated loci and their effector transcripts with known epigenetic regulation discussed in above sections.

## 6. Interactions between Genetics and Epigenetic Control in the Pathogenesis of Type 2 Diabetes

Another strong line of evidence of the interaction between genetics and epigenetics comes from the fact that 25% of all SNPs in the genome either introduce or remove CpG sites. The introduction or removal of CpG dinucleotides according to genotype (termed CpG-SNP) alters the location of potential methylation sites in the DNA sequence [90]. CpG-SNPs have been suggested to be a potential mechanism through which SNPs affect gene function via epigenetics. To date, however, few studies have shown that the association between SNPs and T2D risk occurs through effects on DNA methylation [90,91,92]. The investigation of 40 T2D-associated SNPs revealed that 19 of them (48%) were indeed CpG-SNPs. Additionally, DNA methylation data were generated for 16 of these 19 CpG-SNP loci, representing the *TCF7L2*, *KCNQ1*, *PPARG*, *SRR*, *CHCHD9*, *HHEX*, *ADCY5*, *SLC30A8*, *DUSP9*, *CDKN2A*, *CDKAL1*, *WFS1*, *HMGA2*, *IRS1*, *DUSP8*, and *TSPAN8* candidate genes. Further analyses revealed that some of the CpG-SNPs were associated with gene expression and the secretion of hormones in human pancreatic islets [90]. To identify a wider range of genetic loci that interact with the epigenome in pancreatic islets, genome-wide DNA methylation quantitative trait locus (mQTL) analysis was performed. A total of 574,553 SNPs were compiled with genome-wide DNA methylation data of 468,787 CpG sites. A significant association with methylation at specific CpG sites was found in more than 6% of the analyzed SNPs [91]. Regulation of the promoter methylation state of adiponectin (an adipokine that is involved in regulating insulin sensitivity) is another interesting example of the way in which complex traits, such as obesity, can be linked to CpG-SNPs. In conditions of obesity, DNA methyltransferase 1 is activated in adipose tissue and hypermethylates the adiponectin promoter, leading to a decrease in gene expression. Interestingly, two CpG-SNPs are located within the adiponectin promoter region (rs17300539 and rs266729), which had a significant correlation with serum adiponectin levels [92]. It has been shown that higher circulating adiponectin levels are associated with decreased T2D risk [93].

## 7. Conclusions and Perspectives 

Applying high-throughput methods in diabetes research has accelerated progress in identifying genetic variants and epigenetic modifications that contribute to the pathogenesis of T2D. Technology has developed tremendously, and mathematical and statistical methodologies for data interpretation have improved and become more widely accessible. Importantly, high-throughput-derived data clearly show that environmental factors and exposure to these environmental factors from the prenatal stage to adulthood can lead to (epi)genomic changes that influence the risk of developing T2D. The integration of genetic, epigenetic, transcriptomic and phenotypic information allows to identify genes and novel metabolic pathway targets that deserve further attention to elucidate mechanistic relationships with insulin resistance and pancreatic islet failure. Although the GWASs and EWASs shed light onto (epi)genomic landscape of T2D to a great extent, these methods have still explicit limitations to conquer, such as sample size, small effect size, low allele frequency, genetic heterogeneity and incomplete genotyping. Further research is also needed to track genomic changes in individual pancreatic islet cells as well as additional studies of epigenetic profile of T2D, especially those including histone modifications. Better and more cost-effective “omics” approaches will contribute to a better understanding of T2D and population-oriented treatment. The careful design of population studies and proper validation are crucial for identifying unique (epi)genomic variants that can aid diagnosis and distinguish T2D from other diabetic disorders.

## Figures and Tables

**Table 1:** Rare-coding and non-coding genetic variants implicated in pathogenesis of type 2 diabetes (T2D).

Gene	Chr.	Variant	Type/Location	Risk allele/aa Change	Ethnicity	Pathogenicity	Reference
*HNF1A*	12	chr12:121437091	missense	E508K	US Latino	higher	[40]
*SLC16A11*	17	rs75493593 rs75418188 rs13342692 rs117767867	missense	P443T G40S D127G V113I	European	higher	[40]
*TPCN2*	11	rs1551305	intronic	G	Chinese	higher	[41]
*SLC30A8*	8	8q24.11	missense	R138X	Northern European	reduced	[33]
*CCND2*	12	rs76895963	intronic	G	Icelandic Danish	reduced	[42]
*PDX1*	13	chr13:27396636delT	frameshift	G218Afs*12		higher	
*PAM*	5	rs35658696 rs78408340	missense	D563G S539W		higher	
*THADA*	2	rs7578597	intronic	T	European	higher	[43]
*KCNQ1*	11	rs163184	intronic	G		higher	
*TCF7L2*	10	rs7903146	intronic	T		higher	
*ADRA2A*	10	rs10885122	intronic	G		higher	
*CYB5A*	18	rs7238987	missense	P96P	Pima Indians	higher	[44]
*RNF10*	12	chr12:120990399	missense	R151H		higher	

**Table 2:** T2D associated genetic loci and their transcript genes regulated via epigenetic mechanisms.

T2D loci	Effector Transcript	Epigenetic Signature	Tissue	Approach	Reference Study
*8q24.11*	*SLC30A8*	DMR, open chromatin regions	skeletal muscle, subcutaneous adipose tissue pancreatic islets	DNA methylation array, FAIRE-seq, RRBS	[69,71,90]
*13q12.2*	*PDX1*	open chromatin regions	pancreatic islets	FAIRE-seq	[71]
*4q21.23*	*NKX6.1*				
*10q25.2-q25.3*	*TCF7L2*	DMR	pancreatic islets	WGBS, RRBS	[67,90]
*9p24.2*	*GLIS3*	DMR	pancreatic islets	WGBS	[67]
*2p21*	*THADA*				
*3p25.2*	*PPARG*	DMR	pancreatic islets	RRBS	[90]
*6p22.3*	*CDKAL1*				
*17p13.3*	*SRR*				
*11p15.5*	*DUSP8*				
*12q14.3*	*HMGA2*				
*11p15.5-p15.4*	*KCNQ1*	DMR	skeletal muscle, subcutaneous adipose tissue pancreatic islets	DNA methylation array, WGBS, RRBS	[69] [67] [90]
*10q23.33*	*HHEX*	DMR	skeletal muscle pancreatic islets	DNA methylation array, RRBS	[69] [90]
*3q21.1*	*ADCY5*	DMR	subcutaneous adipose tissue pancreatic islets	DNA methylation array, RRBS	[69] [90]
*Xq28*	*DUSP9*	DMR	skeletal muscle, subcutaneous adipose tissuepancreatic islets	DNA methylation array, RRBS	[69,90]
*9p21.3*	*CDKN2A*				
*12q21.1*	*TSPAN8*	DMR	subcutaneous adipose tissue pancreatic islets	DNA methylation array, RRBS	[69,90]
*4p16.1*	*WFS1*				
*2q36.3*	*IRS1*				
*16q12.2*	*FTO*	DMR	gametes	RRBS	[89]
*16p11.2*	*SH2B1*				
*19q13.11*	*CHST8*				
*1q21.1*	*TXNIP*	DMR	whole blood, liver, pancreatic islets, skeletal muscle	EWAS, WGBS	[80,81]
*21q22.3*	*ABCG1*	DMR	whole blood	EWAS	[80,87]
*17q25.3*	*SOCS3*	DMR	whole blood, liver	EWAS, WGBS	[80,81,87]
*17p11.2*	*SREBF1*		whole blood, pancreatic islets		
*7q32.2*	*KLF14*	DMR	whole blood	WGBS	[81]
*2q12.2*	*FHL2*				
*14q22.1*	*GNPNAT1*				
*11p15.5*	*IGF2*	DMR	whole blood	WGBS	[64]
*20q13.32*	*GNASAS*				
*7q32.1*	*LEP*				
*1q32.1*	*IL10*				
*9q31.1*	*ABCA1*				
*11p15.5*	*INS-IGF2*				
*14q32.2*	*MEG3*				
*3q26.2*	*SLC2A2*				
*9q31.1*	*NR4A3*				
*6q26*	*PARK2*				
*12q21.3*	*SOCS2*				
*2q36.3*	*PID1*				
*4p15.2*	*PPARGC1A*	DMR	skeletal muscle	DNA methylation array	[68,69]
*4q13.3*	*IL8*				[69]
*2p25.1*	*KLF11*				
*20q13.12*	*HNF4A*	DMR	skeletal muscle, subcutaneous adipose tissue	DNA methylation array	[69]
*7q31.2*	*CAV1*		subcutaneous adipose tissue		
*3p25.3*	*CIDEC*				
*9p21.3*	*CDKN2B*				
*10q23.33*	*IDE*				
*11q14.3*	*MTNR1B*				

DMR, Differentially Methylated Region; EWAS, Epigenome-Wide Association Analysis.
